# New Molecular Targeted Therapy and Redifferentiation Therapy for Radioiodine-Refractory Advanced Papillary Thyroid Carcinoma: Literature Review

**DOI:** 10.1155/2012/818204

**Published:** 2012-12-24

**Authors:** Kai-Pun Wong, Brian Hung-Hin Lang

**Affiliations:** Division of Endocrine Surgery, Department of Surgery, The University of Hong Kong, Queen Mary Hospital, Pokfulam Road, Hong Kong, Hong Kong

## Abstract

Although the majority of papillary thyroid carcinoma could be successfully managed by complete surgical resection alone or resection followed by radioiodine ablation, a small proportion of patients may develop radioiodine-refractory progressive disease which is not amenable to surgery, local ablative treatment or other treatment modalities. The use of FDG-PET/CT scan for persistent/recurrent disease has improved the accuracy of restaging as well as cancer prognostication. Given that patients with RAI-refractory disease tend to do significantly worse than those with radioiodine-avid or non-progressive disease, an increasing number of phase I and II studies have been conducted to evaluate the efficacy of new molecular targeted drugs such as the tyrosine kinase inhibitors and redifferentiation drugs. The overall response rate of these drugs ranged between 0–53%, depending on whether the patients had been previously treated with these drugs, performance status and extent of disease. However, drug toxicity remains a major concern in administration of target therapies. Nevertheless, there are also ongoing phase III studies evaluating the efficacy of these new drugs. The aim of the review was to summarize and discuss the results of these targeted drugs and redifferentiation agents for patients with progressive, radioiodine-refractory papillary thyroid carcinoma.

## 1. Introduction

Papillary thyroid carcinoma (PTC) is the most common type of differentiated thyroid carcinoma (DTC) and its age-adjusted incidence has doubled in the last 25 years [1]. Despite its relatively good prognosis with a 10-year cancer-specific survival above 90%, locoregional recurrences and distant metastasis do occur not infrequently [2]. Of the 5–20% patients who may develop locoregional recurrences, approximately two-thirds of these recurrences involved the cervical lymph nodes. On the other hand, up to 10–15% patients would either present with distant metastasis at diagnosis or develop distant metastasis some time after initial treatment [3]. It is not uncommon to encounter patients with initial persistent locoregional recurrence who also later develop distant metastasis. Perhaps, this is a sign of disease progression. Since most patients would have had a total thyroidectomy and radioiodine (RAI) ablation as their initial therapy, disease monitoring or surveillance often relies on regular measurement of thyroglobulin (Tg) and high resolution neck ultrasound (USG) [4]. FDG-PET/CT scan is now often used as a staging tool in patients with suspected disease recurrence. 

In terms of treating locoregional recurrence, a formal selective neck dissection for lymph node recurrence is usually preferred but at times when certain compartments has been previously dissected, a focused neck dissection or completion compartmental neck dissection might be preferred [2]. However, despite the best surgical effort, only approximately one-third of patients would become biochemically cured of the disease (i.e., athyroglobulinemia) and therefore, the American Thyroid Association (ATA) only recommended surgical removal of clinically significant metastatic lymph nodes to prevent future locoregional complications [2, 5, 6]. Other options include percutaneous ethanol injection and radiofrequency ablation (RFA) as their efficacy have been shown in several studies [7–9]. The decision for further adjuvant RAI therapy after reoperative neck dissection depends on the completeness of the dissection [5]. After that, local external beam radiation therapy (EBRT) might be considered in patients with gross unresectable, residual recurrence in the thyroid bed or lateral neck area. Adjuvant external beam radiation in patients with residual microscopic disease could achieve a higher 10-year local relapse-free rate (93% versus 78%) and disease free survival (100% versus 95%) compared with nonradiated patients [10].

In terms of treating patients with distant metastasis, surgical resection is often not the first treatment modality unless a patient has a solitary metastasis which is located near to or in a vital area such as the brain or vertebra. EBRT might be considered in patients with unresectable painful bone metastasis or metastatic lesion which might develop future debilitating complication, for example, fracture, neurological symptoms, compressing or invading of vital structures. In patient with brain metastasis not amendable to surgical resection, whole brain irradiation for multiple lesion or gamma knife radiosurgery for selected patients are acceptable options [5, 11]. RAI is often used as the first-line treatment for patients with distant metastases because it is highly effective in the treatment of small sized distant metastases. Although pulmonary pneumonitis and fibrosis are potential complications which could arise from repeated high-dose RAI treatment, it is recommended that pulmonary micrometastases should be treated with RAI (100–200 mCi) therapy and repeated every 6–12 months so long as the disease continues to concentrate RAI (i.e., RAI-avid) and responds clinically. RAI is generally recommended in patients with nonpulmonary RAI-avid distant metastases, although it might be less effective than pulmonary RAI-avid metastases [2, 5].

**Figure 1:**
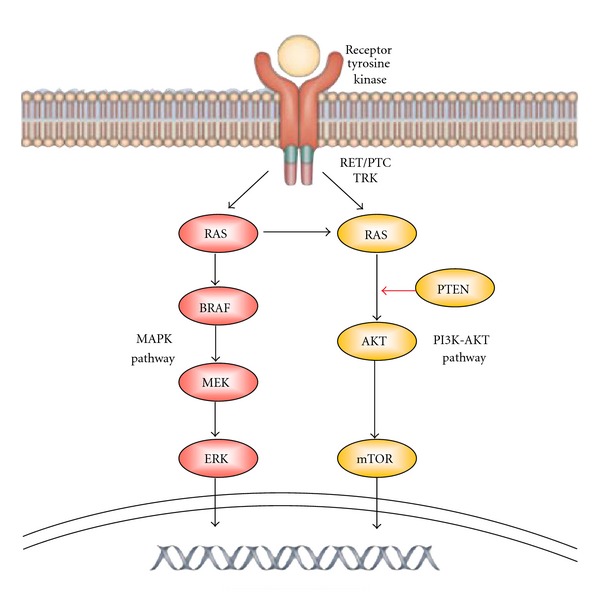
The main signaling pathways involved in thyroid carcinogenesis are the MAPK and PI3K-AKT pathway (reproduced with permission) [15].

As a result, one of the most controversial and difficult issues in treating advanced PTC is how to manage non-RAI-avid disease or RAI-refractory. A patient is defined as having RAI-refractory disease if there is at least one lesion without RAI uptake or a lesion has progressed within a year following RAI treatment or persisted after the administration of a cumulative activity of more than 600 mCi. The current evidence suggests that RAI is of little benefit in patients with RAI-refractory disease [12, 13]. Furthermore, repeated dosage of RAI is associated with dose related complications like salivary gland damage, dental caries, nasolacrimal duct obstruction, and secondary malignancy [5, 14]. Although in the setting of a negative diagnostic RAI scan, small metastases may demonstrate a small measurable benefit to RAI (in terms of decreased Tg), it is of no use at all in large-sized metastases [12, 13]. Therefore, RAI is generally not recommended in RAI-refractory disease and the current treatment options are mainly restricted to conservative treatment, metastectomy, symptomatic control (e.g., drainage of effusions), endobronchial laser ablation, and/or external beam radiation in locally advanced carcinoma. Although age and patient performance status are important considerations when deciding on which treatment option might be preferred, with better understanding and advances in the molecular biology that underlies the development and progression of advanced PTC, many new molecular targeted drugs have been examined or are being examined in the setting of a clinical trial. To date, the available drugs have been targeting two specific molecular pathways in thyroid oncogenesis, namely the mitogen-activated protein kinase (MAPK) pathway and the phosphatidyl-inositol-tri-phosphate kinase (PI3K) pathway.  Figure 1 shows the main signaling pathways involved in thyroid oncogenesis. Redifferentiation therapy is a potential alternative for RAI-refractory disease. Redifferentiating agents could potentially reactivate the RAI uptake ability of DTC. The aim of the present paper is to evaluate the efficacy of these new molecular targeted drugs as well as redifferentiation therapy in the treatment of RAI-refractory advanced or metastatic PTC by reviewing the current literature. 

## 2. Approach to Patients with RAI-Refractory Advanced PTC

Before starting novel therapy, patients with RAI refractory disease should be accurately characterized in terms of age, performance status, histology, disease extent and location, and progression rate [16]. Diagnostic procedures should consist of neck ultrasonography to look for possible concomitant locoregional disease, thin-cut CT neck, thorax, and abdomen to look for other distant metastases and MRI brain for small brain metastases. FDG-PET may also be employed because it helps to localize disease and prognosticate patient risk [36]. For patients with neoplastic foci and high FDG uptakes, local therapy such as radical resection, RFA might be considered as these high FDG-uptake foci are likely to be more aggressive, less differentiated and have higher growth rate [37]. In terms of measuring treatment response, the response evaluation criteria in solid tumors (RECIST) criteria are often used. This is carried out by repeating standardized imaging (usually CT or MRI) every 6 months. Interestingly, there is no evidence to suggest that novel treatment such as targeted therapy should be better given at an early stage than a later stage when the tumor might be larger in size. As most patients with RAI-refractory disease might be asymptomatic for a long period of time, the benefits of targeted therapy must not be outweighed by the drug toxicities and side effects. Therefore, targeted drug is usually commenced when there is documented disease progression by standardized imaging. 

## 3. Potential Molecular Targets

Figure 1 shows the main signaling pathways involved in thyroid oncogenesis [15]. Since rearrangements of *RET* (or *RET/PTC*) and point mutations of *RAS* and *BRAF* are now believed to be initiating events in the carcinogenesis of PTC, most new targeted drugs have the ability of inhibiting the MAPK pathway and angiogenesis. *RET/PTC * rearrangements are found more frequently in classical *PTC* whereas *RAS* point mutations are mostly found in the follicular variant of PTC. BRAF mutations are mostly found in less differentiated or tall-cell variant of PTC. The PI3 K pathway may also be activated in some PTC. Other potential targets would be the angiogenic factors like various vascular endothelial growth factors (VEGF 1 and 2), fibroblast growth factor (FGF), and platelet-derived growth factor (PDGF). In vitro studies showed that anti-VEGF therapy could delay DTC growth [35]. Table 1 shows the relationship between various targeted drugs and their molecular targets.

## 4. New Molecular Targeted Therapy

Table 2 lists the results of various published trials for new targeted therapy in RAI-refractory thyroid carcinoma. Sorafenib was one of the first agents studied in RAI refractory PTC. It is an oral multi-tyrosine kinase inhibitor with multiple targets, including *VEGF-R* 1 to 3, *PDGF-R*, *RET/PTCs*, and *BRAF*. Two American studies have published the results of using sorafenib 400 mg twice daily in advanced PTC [19, 20]. Gupta-Abramson et al. reported the first phase II study in advanced PTC. Of the 18 patients with advanced PTC who received treatment for a minimum of 16 weeks, 4 out of 18 patients had partial response and 10 had stable disease. In this study, the median progression-free survival was 84 weeks [19]. Kloos et al. reported another phase II study on 41 patients with metastatic papillary thyroid cancer. Of these patients, 15% had partial response and 56% had stable disease lasting for more than 6 months. The median progressive free survival was 15 months [20]. Adverse events were common, most frequent being fatigue (60–74%), hand foot syndrome (58–83%), diarrhea (68–73%), and muscle pain (28–57%) [19, 20]. Most of them were grade 1 or 2, and could be managed with dose reduction or drug holiday. It is interesting to note that about half the patients had to have dose reduction to improve compliance and control toxicity. Total withdrawal of treatment due to grade 3 or worse adverse events were not uncommon (about 16.6%) [19]. Since then, 3 other studies from Europe were published. In a Netherland's study of 30 patients with DTC, 25% of patients achieved partial response. The response rate did not appear to be influenced by gender, age, initial stage, or presence of *BRAF* mutation. However, the radiological response was worse in patients with bone metastases [21]. The authors also studied the possibility of reinduction of RAI uptake after starting the drug but no reinduction was observed [21]. A UK study evaluated 19 patients with DTC treated with sorafenib 400 mg twice daily and the partial response was 18% at 12 months [23]. Another multicenter study conducted in Spain on metastatic DTC reported the response rate in DTC and PTC were 19% (3 out of 16 patients) and 14% (1 out of 7 patients), respectively. The median progression free survival was 13.5 months in patients [24]. These results appeared comparable to those reported in the two US studies.

In view of the drug toxicities, some authors attempted to reduce the standard dose and evaluated its efficacy. Chen et al. evaluated the efficacy of low dose sorafenib (200 mg twice daily) on 9 patients with RAI refractory pulmonary metastases. After three months of treatment, the partial response (by RECIST criteria) was 33% and stable disease was achieved in 44% of patients. All adverse events were grade 1 or 2. From this study, it appeared that low dose sorafenib could equally achieve satisfactory response in RAI-refractory PTC [22]. Based on initial success in sorafenib, a phase III international randomized controlled trial is currently underway to evaluate it in progressive RAI-refractory metastatic DTC. In this trial, patients are randomized into the placebo arm and drug arm and the primary endpoint is progression-free survival. The final results are awaiting. (NCT00984282).

Axitinib is an oral, potent selective inhibitor of *VEGFRs* 1, 2, and 3. It is selectively less potent in inhibiting platelet derived growth factor receptor beta and *c-KIT*. Cohen et al. published a phase II study on 60 patients with advanced thyroid cancer. Thirty patients with PTC received axitinib 5 mg twice daily oral. Eight patients (26.7%) had partial response. Stable disease lasting for more than 16 weeks was observed in 12 patients (40%). Overall, the median progression-free survival was 18.1 months. Thirty-two patients discontinued axitinib treatment either because of lack of efficacy (10 patients), or adverse effect (8 patients). Hypertension, proteinuria, and fatigue were most common grade 3 or worse events [25].

Motesanib (AMG-706) is a novel oral inhibitor of multiple tyrosine kinases, including *VEGF* receptors, *PDGF* and *KIT*. In an open label phase 2 trials, 93 patients with progressive, locally advanced, or metastatic, RAI-refractory DTC were prescribed with 125 mg motesanib once daily orally. Fifty-seven (61%) patients had PTC. The objective radiological response assessed was 14%. Of these, 67% achieved stable disease while 35% maintained stable disease for 24 or more weeks. In 75 patients whom had Tg assay, 61 (81%) had reduction in Tg levels. The authors found a significant correlation between the drop in baseline Tg and radiological response rate. Similar to other drugs, adverse effects were a major concern. 94% of all patients experienced adverse events, and 62% had grade 3 or above adverse events. The most common adverse events were hypertension (25%) or diarrhea (13%) There were 2 treatment-related deaths; both were due to pulmonary hemorrhage [26].

Sunitinib is a multitargeted tyrosine kinase inhibitor of *VEGFR* type 1 and 2, *PDGFR∂*, and *β*, *c-Kit*, *FLTF*, and *RET*. In a phase II study, 37.5 mg daily sunitinib was prescribed continuously to 35 patients with FDG-avid, RAI-refractory DTC or medullary thyroid carcinoma (MTC). 28% of patients with DTC achieved a RECIST response assessed by 3 monthly CT scan. The median time to progress was 12.8 months in this series. The functional response was also analyzed in this study. In 19 patients with DTC who underwent PET/CT scan before and after 7 days of sunitinib therapy, the percentage change of SUV were −13.5%, 17.5%, and 9.0% for patients with RECIST response, with stable disease and with progressive disease respectively. There was a significant correlation between SUV changes and changes in RECIST criteria (*P* = 0.005). Despite satisfactory functional and RECIST response, hematological and other treatment-related adverse events were also common. Upto 34% of patients suffered grade 3 or worse hematological side effects including leukopenia, neutropenia. Other common adverse effects (≥grade 3) including diarrhea (17%), hand foot syndrome (17%), and fatigue (11%) [27]. Cohen et al. reported another series of 43 patients with either DTC or MTC. In 37 patients with DTC, response rate was 13% and 68% had stable disease. Grade 3/4 haematological adverse effect were comparably common [28].

Pazopanib (Votrient) is a tyrosine kinase inhibitor targeting *VEGFR*, *PDGF*, and *c-KIT*. It has an antiangiogenic effect on RAI-refractory DTC. In a phase II study on 37 patients with progressive radioiodine refractory DTC over previous 6 months, partial response was achieved in 18 patients. Response rate was 49% and 33% (5 out of 15%) in patients with DTC and PTC, respectively. 66% of patients were likely to respond lasting for more than 1 year. The response rate was highest among reports of different agent. Adverse effects were common but generally well tolerate. Grade 3 or worse adverse effect was not common, and the commonest was deranged liver function tests (4 patients had raised ALT concentration). Two patients had serious hemorrhagic events (grade 3 lower gastro-intestinal bleeding, and grade 4 intracranial bleeding in the absence of brain metastasis or hypertension) and so had to discontinue treatment [29].

Lenvatinib (E7080) is an oral tyrosine kinase inhibitor targeting *VEGFR* 1–3, *FGFR* 1–4, *RET*, *KIT*, and *PDGFRβ*. In an international phase II study, 58 patients with advanced progressive DTC were enrolled. Half of them achieved partial response on assessment. For the subgroup which had prior *VEGFR* inhibitors, 41% had a response whereas those without prior treatment, 54% had a response. However, because adverse effect was common, 23% of patients had to withdraw and 35% had to have a dose reduction [30]. 

Cabozantinib (XL84) is an oral potent inhibitor of *cMET* (MET is a membrane receptor that is essential for embryonic development and wound healing), *VEGFR2*, and *RET* and is currently undergoing a clinical trial. The results of the phase I trial of this drug appeared promising with 8/15 (53%) patients having confirmed partial response and 6 (40%) had stable disease [31]. It is worth noting that the majority of these patients had prior *VEGFR* inhibitors. Grades 3, and 4 adverse effects were comparable to other *VEGFR* inhibitors and included diarrhea (20%), hypertension (13%), and hand foot syndrome (13%) [31].

Vandetanib, a tyrosine kinase inhibitor of *RET*, *VEGFR*, and *EGFR* signaling, was the first targeted therapy subjected to large scale multicenter randomized clinical trial [32]. 145 patients with radioiodine refractory differentiated thyroid cancer were enrolled into the trial. Seventy-two patients, including 25 with PTC, were allocated to vandetanib 300 mg per day group and 73 patients, including 24 with PTC were allocated to placebo group. Patients in vandetanib group (median: 11.1 months (95% CI 7.7–14.0)) had longer progression-free survival than placebo group [median: 5.9 months (95% CI 4.0–8.9) (*P* = 0.017). For patient with PTC, median progression-free survival was 16.2 months (95% CI 8.4–22.6) in vandetanib group and 5.9 months (95% CI 3.0–11.5) in placebo group. Although it was not statistically significant, there was a tendency for clinical benefit (Hazard ratio: 0.52, 95% CI 0.26–1.02, *P* = 0.056). However, there was no difference in overall survival (Hazard ratio: 0.83, 95% CI 0.52–1.33, *P* = 0.42). Most frequent adverse events were diarrhea (74%) and hypertension (34%). The incidence of grade 3 or worse adverse events were higher in vandetanib group (53%, 39/73 patients) than placebo group (19%, 14/72 patients), while the commonest were QTc prolongation (14%) and diarrhea (10%) [32].

Since *EGFR* mutations were found in some PTC, Gefitinib, an inhibitor of *EGFR* tyrosine kinase, had been evaluated in a phase II study for advanced PTC. Pennell et al. reported the results of 27 patients with 11 of them suffering from PTC. There was no objective response observed. 24% had stable disease after 6 months of treatment. Five patients with stable disease had significant drop in thyroglobulin (>90% drop) maintained over 3 months. However, no patient achieved partial response by criteria. The authors concluded that this drug had limited biological activity of around 12% only [33].

Other than antiangiogenic therapies, drugs targeting specific molecular pathway, namely MAPK and PI3K, were studied in phase I, and II studies. Selumetinib is a selective oral non-ATP competitive small molecule inhibitor of *MAPK* kinases, *MEK* 1/2. In in vitro studies, Selumetinib was potent in PTC cell line with V600E *BRAF* mutation. It had been recently evaluated in a multicenter phase II study in patient with radioiodine refractory papillary thyroid carcinoma. Selumetinib 100 mg twice daily was prescribed to patients with document progression in the last 12 months. Only 1 patient (3%) achieved partial response and 21 (54%) had stable disease. Common grade 3 to 4 toxicities included rash (18%), fatigue (8%), diarrhea (5%), and peripheral edema (5%). In *BRAF* evaluated patient, *BRAF* mutant had a longer median progression free survival than *BRAF* wide-type tumor, though it was not statistically significant (33 versus 11 weeks, *P* = 0.3) [34].

In a recent published phase I trial, Dabrafenib, inhibitor of *BRAF* kinase for *BRAF* mutant, had showed its anti-tumor activity in 14 patients with *BRAF* mutant PTC. Three of nine assessable patients achieved partial response. Its adverse effect was less common and more tolerable. The most common grade 2 or worse adverse effects were cutaneous squamous cell carcinoma (11%), fatigue (8%), and pyrexia (6%) [35].

In addition to agents which target MAPK and PI3 K-AKT pathways, *mTOR* inhibitors have been widely studied recently. In vitro studies, *mTOR* pathway activation have been found in aggressive and *BRAF* mutated PTC [38, 39]. Phase I study of daily everolimus plus low dose weekly cisplatin reported 1 out of 4 patients achieved prolong stable disease for 6 months [40]. Sherman et al. reported phase II study on use of combination of sorafenib and temsirolimus in 37 patients radioiodine refractory thyroid carcinoma. 21.6% patients had partial response and 56.7% had stable after median time on treatment of 206 days [41].

Studies on combination of target therapy like everolimus/lenalidomide, pazopanib/GSK1120212 (*MEK* inhibitor), and pimasertib (*MEK* inhibitor)/SAR245409 (PI3K/*mTOR* inhibitors) are ongoing and hopefully, results would become available in the near future [42–44].

Majority of studies focused on patients with PTC or small subgroup of follicular thyroid carcinoma (FTC) patients (Table 2); current evidences on treatment specific on FTC were limited. Applicability and efficacy of target therapies on patient with follicular thyroid carcinoma have to be further evaluated.

## 5. Redifferentiation of RAI-Refractory Thyroid Carcinoma

Redifferentiation remains an ongoing area of research in RAI-refractory thyroid carcinoma because development of de-differentiated phenotype of DTC leads to loss or absence of radioiodine uptake ability. Redifferentiating agents could potentially reactivate the RAI uptake ability of thyroid carcinoma. Retinoids, chemically related to Vitamin A, were the first widely studied agents for potential application on redifferentiation in radioiodine refractory thyroid cancer.

Early in vitro studies showed that decreased expression of retinoic acid receptor in thyroid cancer cell [45]. Treatment with retinoids could upgrade the sodium-iodine symporter (NIS) and thus induce redifferentiation and iodine uptake by tumor cell. Grüning et al. reported their series on 14 RAI-refractory patients and showed that 2/14 showed slightly increased uptake [46]. Similar results have been found by other authors. Short et al. reported the results of 16 patients with RAI uptake negative DTC. Patients were prescribed oral isotretinoid 1.5 mg/kg/day for 8 weeks. RAI scan repeated 2 weeks later reviewed only 1/16 (6.25%) patient had increased radio-iodine uptake [47]. Simon et al. reported a more promising result of using 13-cis-retinoic acid as a sole treatment. Fifty patients with RAI-refractory DTC were treated with 13-cis-retinoic acid at dosage of 1.5 mg/kg daily over 5 weeks. Thirteen (26%) showed marked increase in RAI uptake while 8 patients (16%) showed mild increased in RAI uptake in the post-therapy scan. Tumor size was assessed in 37 patients and of these, 6 had tumor regression while 22 had static disease. As a whole, 19 (38%) patients showed either response or stable in clinical response. However, further analysis reviewed that increased RAI uptake did not always correlate with clinical response [48]. More recently, Oh et al. incorporated retinoic acid into RAI ablation to treat RAI refractory PTC. Oral isotretinoid was prescribed for 6 weeks at 1–1.5 mg/kg daily and then followed by single dose of RAI. At 6-month follow up, 1 patient showed completed response, 9 had partial response, 9 had stable and 28 had progressive disease. Overall response rate was 21.3% [49].

Due to the limited benefits, routine use of retinoic acid alone is not recommended. Preliminary study showed enlightening clinical response in refractory cancer results only in combination therapy. Therefore, further studies are indicated to evaluate its clinical application, dosage, duration, and combination regimen. 

Thiazolidinedione, an antidiabetic medication, is another potential redifferentiation agent. It acts as a peroxisome proliferator-activated receptor gamma agonist. It was shown to have both redifferentiation and anti-proliferative effects in in vitro study of thyroid cancer cells [50]. In an open phase II trial of 20 patients with Tg-positive RAI-refractory DTC, 25% (5/20) regained RAI uptake. 60% (12/20) had stable disease and 15% (3/20) had reduction in tumor marker. Overall, 5 patients had a partial response in terms of decreased Tg or positive RAI uptake on scan. However, no complete or partial response was noted in any patients according to RECIST criteria [51]. Other agents, including histone deacetylase inhibitor, (e.g., valproic acid, trichostatin, and depsipeptide), DNA demethylating agents, (e.g., 5-azacytidine), and arsenic trioxide, had shown differentiation inducing properties in thyroid cancer cells in in vitro studies [52–55]. Potential clinical application had to be further validated by future studies. 

## 6. Conclusion

Managing RAI-refractory advanced or metastatic PTC remains challenging. Given the fact that RAI is of little benefit in this group of patients, various treatment options such as observation, symptomatic control, surgery, local ablative therapy, metastectomy, and EBRT might be considered. New molecular targeted drugs including tyrosine kinase inhibitors and redifferentiation drugs are new promising therapeutic options for this specific group of patients. 

## Figures and Tables

**Table 1:** Kinase inhibitor activities of target therapies on thyroid cancer [16–18].

Drug	Inhibitory concentration 50 [IC_50_] (nmol/L)							
	VEGFR-1	VEGFR-2	VEGFR-3	RET	BRAF^V600E^	PDGFR-*β*	Kit	Others
Sorafenib	26	90	20	49	25	57	68	
Axitinib	1.2	0.25	0.29			2	1.7	
Motesanib	2	3	6	59		84	8	
Sunitinib	2	9	17	41		39	1–10	
Pazopanib	10	30	47			84	74	
Lenvatinib (E7080)	22	4	5	35		39		FGFR1 (46)
Cabozantinib (XL-184)		0.035		4				cMET (1.8)
Vandetanib	1600	40	110	130				EGFR (500)
Gefitinib		>10000		3700				EGFR (33)
Selumetinib								MEK1 (14)
Dabrafenib					0.8			

**Table 2:** Study on target therapy in radioiodine-refractory papillary thyroid cancer.

Author	Study	Agent	Number of patients [type]	Result			
				Response rate (%)	Stabilization (%)	Progression-free survival (months)	Discontinuation due to adverse effect
Gupta-Abramson et al. [19]	Phase II	Sorafenib	30 [DTC and MTC] (i) DTC: 27/30 (ii) PTC: 18/30	23% 25.9% 22.2%	53% 55.6% 61.1%	18.2 (79 weeks) 19.3 (84 weeks)	16.7%

Kloos et al. [20]	Phase II	Sorafenib	41 [PTC]	15%	56% (≥6 months)	15	—

Hoftijzer et al. [21]	Phase II	Sorafenib	31 [DTC] PTC: 14/31	25%	34% (26 weeks)	13.3 (58 weeks)	19.4%

Chen et al. [22]	Phase II	Sorafenib	9 [PTC]	33%	44%	9.6 (42 weeks)	0

Ahmed et al. [23]	Phase II	Sorafenib	34 [MTC and DTC] PTC: 19/34	15% 18%	74% (6 months)	—	5.9%

Capdevila et al. [24]	Phase II	Sorafenib	34** (i) DTC: 16/34 (ii) PTC: 7/34	32% 19% 14%	41% (6 months) 50% 43%	13.5	—

Cohen et al. [25]	Phase II	Axitinib	60 [DTC] PTC: 30/60	30% 26.7%	38% (≥16 weeks) 40%	18.1	13%

Sherman et al. [26]	Phase II	Motesanib	93 [DTC] PTC: 57/93	14%	35% (24 weeks)	9.3	13%

Carr et al. [27]	Phase II	Sunitinib	35 [DTC and MTC] (i) DTC: 28/35 (ii) PTC: 18/35	31% 28%	46% 68%	12.8	11.4%

Cohen et al. [28]	Phase II	Sunitinib	31 [DTC]	13%	68%	—	—

Bible et al. [29]	Phase II	Pazopanib	37 [DTC] PTC: 15/37	49% 33%	—	11.7	5.4%

Sherman et al. [30]	Phase II	Lenvatinib	58 [DTC] PTC: 43/58	50%	36%	13.3	23%

Cabanillas et al. [31]	Phase II	Cabozantinib	15 [DTC]	53%	40%	—	—

Leboulleux et al. [32]	RCT, phase II	Vandetanib	72 versus 73 [DTC] PTC: 25 versus 24	8.3% versus 5.5%	56% versus 36%*	11.1 versus 5.8* 16.2 versus 5.9	33% versus 6%

Pennell et al. [33]	Phase II	Gefitinib	27** PTC: 11/27	0%	24% (6 months)	3.7	7.4%

Hayes et al. [34]	Phase II	Selumetinib	32 [PTC]	3%	36% (24 weeks)	7.4	—

Falchook et al. [35]	Phase I	Dabrafenib	14 [PTC]	21.4%	—	—	0

DTC: differentiated thyroid cancer, PTC: papillary thyroid cancer, MTC: medullary thyroid cancer; RCT: randomized controlled trial.

*P < 0.05.

**Included differentiated, medullary, and anaplastic thyroid carcinoma.
